# Plasma ^1^H-NMR metabolic and amino acid profiles of newborn piglets from two lines divergently selected for residual feed intake

**DOI:** 10.1038/s41598-023-34279-5

**Published:** 2023-05-02

**Authors:** Laurence Liaubet, Camille Guilmineau, Gaëlle Lefort, Yvon Billon, Sébastien Reigner, Jean Bailly, Nathalie Marty-Gasset, Laure Gress, Rémi Servien, Agnès Bonnet, Hélène Gilbert, Nathalie Vialaneix, Hélène Quesnel

**Affiliations:** 1 GenPhySE, Université de Toulouse, INRAE, ENVT, 31326 Castanet Tolosan, France; 2 Université de Toulouse, INRAE, UR MIAT, 31326 Castanet-Tolosan, France; 3 INRAE, GENESI, 17700 Saint Pierre d’Amilly, France; 4 INRAE, Univ. Montpellier, LBE, 102 Avenue des étangs, 11100 Narbonne, France; 5 PEGASE, INRAE, Institut Agro, 35590 Saint-Gilles, France; 6 CNRS, IFCE, INRAE, Université de Tours, PRC, 37380 Nouzilly, France

**Keywords:** Developmental biology, Physiology, Systems biology, Animal breeding, Functional genomics, Genetics, Development

## Abstract

Together with environmental factors, physiological maturity at birth is a major determinant for neonatal survival and postnatal development in mammalian species. Maturity at birth is the outcome of complex mechanisms of intra-uterine development and maturation during the end of gestation. In pig production, piglet preweaning mortality averages 20% of the litter and thus, maturity is a major welfare and economic concern. Here, we used both targeted and untargeted metabolomic approaches to provide a deeper understanding of the maturity in a model of lines of pigs divergently selected on residual feed intake (RFI), previously shown to have contrasted signs of maturity at birth. Analyses were conducted on plasma metabolome of piglets at birth and integrated with other phenotypic characteristics associated to maturity. We confirmed proline and myo-inositol, previously described for their association with delayed growth, as potential markers of maturity. Urea cycle and energy metabolism were found more regulated in piglets from high and low RFI lines, respectively, suggesting a better thermoregulation ability for the low RFI (with higher feed efficiency) piglets.

## Introduction

Together with environmental factors, physiological maturity at birth is a major determinant for neonatal survival and postnatal development in mammalian species^1,2^. Maturity at birth can be defined as a complete development enabling adaptation to extra-uterine life. It is the outcome of complex mechanisms of intra-uterine development and maturation during the end of gestation^1,2^. In pig production, piglet preweaning mortality averages 20% of the litter. Thus, it is a major welfare concern and such a mortality also causes economic loss^3^. A deeper understanding of piglet maturity at birth may help provide tools to effectively reduce neonatal mortality. In the present study, piglet maturity at birth was explored by using a model of lines divergently selected on residual feed intake (RFI). After ten generations of selection, piglets are lighter at birth and grow faster during lactation in the line selected for low RFI (LRFI, i.e. the most efficient animals in terms of RFI)^4,5^. Moreover, a difference between lines was observed in piglet thermoregulation abilities using infrared thermography, with LRFI piglets showing a quicker increase in ear tip temperature within one hour after birth than HRFI piglets^6^. Together, these findings suggest that piglet maturity differs between these lines.

A metabolomic approach by ^1^H Nuclear Magnetic Resonance (NMR) has proven to be effective in studying the maturity of pig fetuses^7^. Maturity was explored by comparing two stages at the end of gestation, 90 and 110 days (birth is expected at 115 days), and two breeds (Large White and Meishan) that strongly differ in terms of neonatal survival. Findings suggested that the biological processes related to the amino acid, carbohydrate, and glutathione metabolisms were involved in piglet maturity. They also confirmed that proline and myo-inositol could be good markers of maturity and associated with delayed growth. Moreover, the delayed maturity of Large White fetuses when compared to Meishan fetuses has been confirmed at the metabolic level while it had already been observed with other omics analyses^8–11^ just before birth (transcriptomic and proteomic profiles of muscular, intestinal, and adipose tissues).

In this context, the present study aimed, first, at comparing the plasma metabolome of LRFI and HRFI piglets at birth, and second, at investigating relationships between plasma metabolites and piglet phenotypes that may be related to piglet maturity at birth (birth weight, morphological traits, and rectal temperature). Given the importance of amino acid (AA) metabolism in maturity^7,12^, the focus was given to AA by coupling the Ultra Performance Liquid Chromatography (UPLC) approach to the metabolomics approach.

## Results

Ninety-seven piglets were involved in this study. Piglets were studied just after birth, before any suckling. Biometric information was body weight (Weight), crown-to-rump length (Length), width between the two shoulders (Width), chest circumference (Circ), rectal temperature (Temp), calculated body mass index (BMI), and ponderal index (PI). Umbilical cord plasma was sampled for an untargeted ^1^H-NMR analysis together with a targeted analysis of 20 proteinogenic amino acids (AA) and 7 other compounds (non-proteinogenic AA and dipeptides) by UPLC. Results are detailed in Supplementary Table S1.

### Identification and quantification of metabolites

Automatic identification and relative quantification of metabolites from NMR spectra were performed with the R package ASICS^12,13^. From a library of pure spectra of 190 metabolites, 72 metabolites were quantified including 15 AA in common with those quantified by UPLC. Compared to the AA analyzed by UPLC, four AA (histidine, lysine, threonine, and tryptophan) were not quantified by ASICS while they were present in the library of pure spectra. ^1^H-NMR is often considered as a less sensitive technology for metabolomics than mass spectrometry^14^; some metabolites cannot be detected, especially when they are in low concentration. This could be the case of tryptophan with low mean values when measured by UPLC (Supplementary Table S1). Histidine, lysine, and threonine were between 5 and 15 times more concentrated in the plasma than tryptophan, and further investigations are needed to understand why they were not detected by ASICS. Similarly, we did not detect histidine, lysine, and tryptophan in plasma of 90- and 110-day-old pig fetuses^12^. Moreover, cysteine was detected by UPLC while the ^1^H-NMR method detected the cystine, a form composed of two cysteine molecules and described as more stable than cysteine.

Correlations between AA concentrations obtained by UPLC and those obtained by 1H-NMR/ASICS are presented in Table 1. For the 14 proteinogenic AA that could be assayed by both methods, significant correlations were observed consistently with our previous findings^12^.

**Table 1:** Correlations between plasma proteinogenic amino acid concentrations in newborn piglets measured by UPLC and those measured by ^1^H NMR and quantified with the R package ASICS.

	Spearman's rank correlation coefficient	*p* value
Alanine	0.91	2.5e−15
Arginine	0.74	1.1e−08
Asparagine	0.59	7.5e−09
Aspartate	Only by NMR	
Cysteine	Only by UPLC	
Cystine	Only by NMR	
Glutamate	0.79	3.3e−15
Glutamine	0.91	2.7e−23
Glycine	0.84	2.2e−11
Histidine	Only by UPLC	
Isoleucine	0.58	4.1e−05
Leucine	0.77	7.1e−07
Lysine	Only by UPLC	
Methionine	0.72	2.1e−13
Phenylalanine	0.84	1.7e−17
Proline	0.39	0.0213
Serine	0.76	1.4e−18
Threonine	Only by UPLC	
Tryptophan	Only by UPLC	
Tyrosine	0.89	1.6e−22
Valine	0.82	1.5e−09

The complete dataset included 83 metabolites, i.e., 27 AA and 56 metabolites from ^1^H-NMR/ASICS. In order to have a specific focus on AA and avoid redundancy in the subsequent analyses, the AA dataset was kept from the UPLC results and AA were discarded from the NMR dataset except for cystine and aspartate (identified only in the NMR dataset).

### Identification of influential traits for the genetic line

Supplementary Table S1 (detailed results) and Table 2 (summarized results) provide results on metabolites found influential (VIP > 1) in the Orthogonal Partial Least Squares Discriminant Analysis (OPLS-DA) and on metabolites with significant differences in quantification between the two lines (Wilcoxon adjusted *p*-value < 0.05). In addition, parameters of the OPLS-DA are provided in Supplementary Table S3.

**Table 2:** Metabolites influential between the two RFI divergent lines at birth. Variables are declared influential according to their VIP values for differentiating the two lines (VIP > 1) and their adjusted *p*-value for the Wilcoxon test (adjusted *p* value < 0.05, details in Supplementary Table S1). *AA/UPLC* amino acids assayed by UPLC, *ASICS/NMR* metabolites assayed by NMR and quantified with ASICS.

	Number of influential variables	LRFI > HRFI		LRFI < HRFI	
AA/UPLC	7	1	Tryptophan	6	Arginine, asparagine, histidine, lysine, ornithine, taurine
ASICS/NMR	18	3	Lactate, methanol, pyruvate	15	2-Oxoglutarate, 2-picolinic acid, adenosine, aspartate, betaine, cadaverine, gamma-aminobutyric acid, homovanillic acid, isovaleric acid, kynurenic acid, n-acetyl-l-aspartic acid, pantothenic acid, quinolinic acid, s-acetamidomethylcysteine, trans-ferulic acid

**Figure 1:**
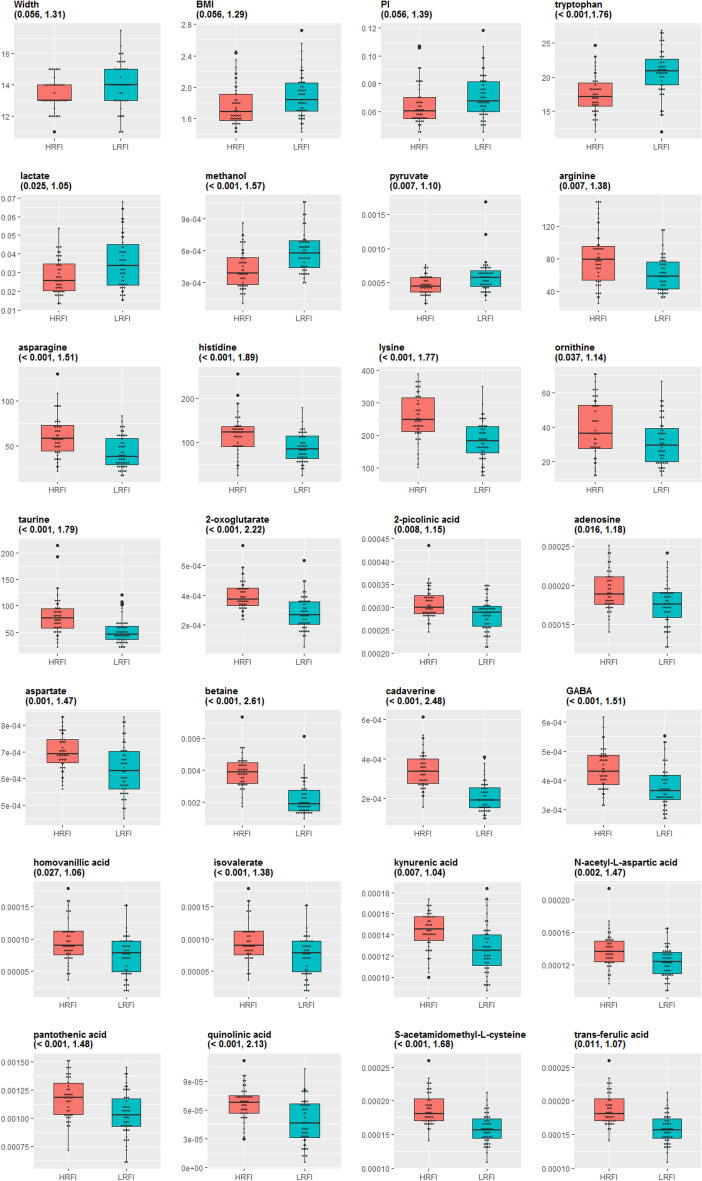
Main phenotypic and metabolic differences between the two genetic lines. The Wilcoxon adjusted *p*-value and the score of the VIP for the analysis of the line are given between brackets.

OPLS-DA results identified piglet shoulder width, BMI, and PI as influential phenotypes for the genetic line (VIP values > 1; Supplementary Table S1) even if these phenotypes were not found differential between the two lines (Wilcoxon adjusted *p*-value = 0.056, Fig. 1). Similarly, seven AA were found influential (Table 2, Fig. 1). Tryptophan was more concentrated in the plasma of LRFI piglets while arginine, asparagine, histidine, lysine, ornithine, and taurine were more concentrated in HRFI piglets (details in Supplementary Table S1). Among the 56 metabolites quantified with ASICS from ^1^H-NMR spectra, 18 were found to be both differential and influential (Table 2, Fig. 1). Three metabolites (lactate, methanol, and pyruvate) were found more concentrated in the LRFI line, while the 15 other influential metabolites were found more concentrated in HRFI piglets. These results were confirmed by Wilcoxon tests but not by mixed models including a random sow effect nested in the line effect (Supplementary Table S1).

### Metabolic pathways related to the genetic line

Enrichment analysis was performed on all the 25 most important metabolites (the 18 found influential and differential in the ASICS dataset and the 7 found influential in the AA dataset), with the online MetaboAnalyst 5.0. The top enriched pathways are presented in Table 3.

**Table 3:** Result of the metabolomics pathway enrichment analysis. Top enrichment analysis with (A) all differential metabolites, (B) influential metabolites more abundant in more efficient LRFI piglets, and (C) influential metabolites more abundant in less efficient HRFI piglets. a: Total number of metabolites referenced in the pathway in the database, b: number of influential metabolites in this study, c: False Discovery Rate (adjusted *p*-value for the control of the FDR).

Pathways	Total number of metabolites^a^	Hits^b^	*p* value	FDR^c^
A. All influential metabolites				
Urea cycle	29	5	1.13E−4	0.00903
Ammonia recycling	32	5	1.84E−4	0.00903
Aspartate metabolism	35	5	2.87E−4	0.00937
Beta-alanine metabolism	34	4	0.00276	0.0654
Glycine and serine metabolism	59	5	0.00334	0.0654
Glutamate metabolism	49	4	0.0106	0.17
Malate-aspartate shuttle	10	2	0.0134	0.17
Arginine and proline metabolism	53	4	0.0139	0.17
Glucose-alanine cycle	13	2	0.0225	0.217
Tryptophan metabolism	60	4	0.0213	0.217
Gluconeogenesis	35	3	0.0244	0.217
B. LRFI > HRFI				
Gluconeogenesis	35	2	0.00333	0.298
Pyruvate metabolism	48	2	0.00627	0.298
Warburg effect	58	2	0.00912	0.298
C. HRFI > LRFI				
Aspartate metabolism	35	5	1.16E−4	0.0114
Urea cycle	29	4	7.48E−4	0.0341
Ammonia recycling	32	4	0.0011	0.0341
Beta-alanine metabolism	34	4	0.00139	0.0341

**Figure 2:**
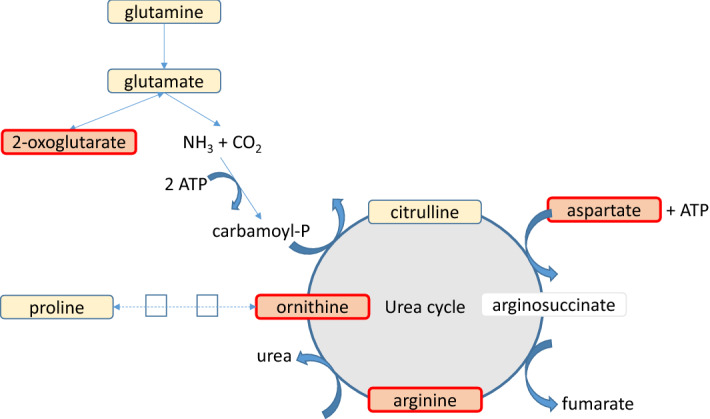
Urea cycle upregulated in piglets from the HRFI line. This network summarizes metabolic pathways linking the urea cycle with proline synthesis and glutamine-oxoglutarate metabolism. The red box indicates the metabolites with significantly higher concentrations in piglets from the HRFI line. The yellow box indicates identified but not differential metabolites.

Among metabolites, 2-oxoglutarate, arginine, aspartate, and ornithine were significantly more concentrated in piglets from the HRFI line and involved in the urea cycle, proline synthesis, and glutamine-oxoglutarate metabolism. These three pathways were manually joined, which mainly highlighted the urea cycle as differentiating the two lines (Fig. 2).

### Multivariate modeling of biometric phenotypes, mortality, and rectal temperature

The seven traits (Weight, Length, Width, Circ, Temp, BMI, and PI) were analyzed by OPLS modeling as numeric variables (Supplementary Tables S2 and S3) and the mortality within 5 days after birth was explored by OPLS-DA as a binary trait. Fourteen piglets born alive (14%) died before weaning, including four in the hours following birth, six on the first day after birth, two on the second day, one on the third day, and one on the fifth day. OPLS-DA allowed identifying possible influential metabolites (even with VIP > 2); such as glycine, pyroglutamic acid, and serine for premature death (within 5 days after birth), betaine and quinolinic acid for width at shoulder, carnosine, histidine, NADP, and 4-aminohippuric acid for BMI, 4-aminohippuric acid also for PI, hydroxy-proline and pantothenic acid for rectal temperature.

### Multivariate analyses to identify covariations between metabolites and phenotypes

**Figure 3:**
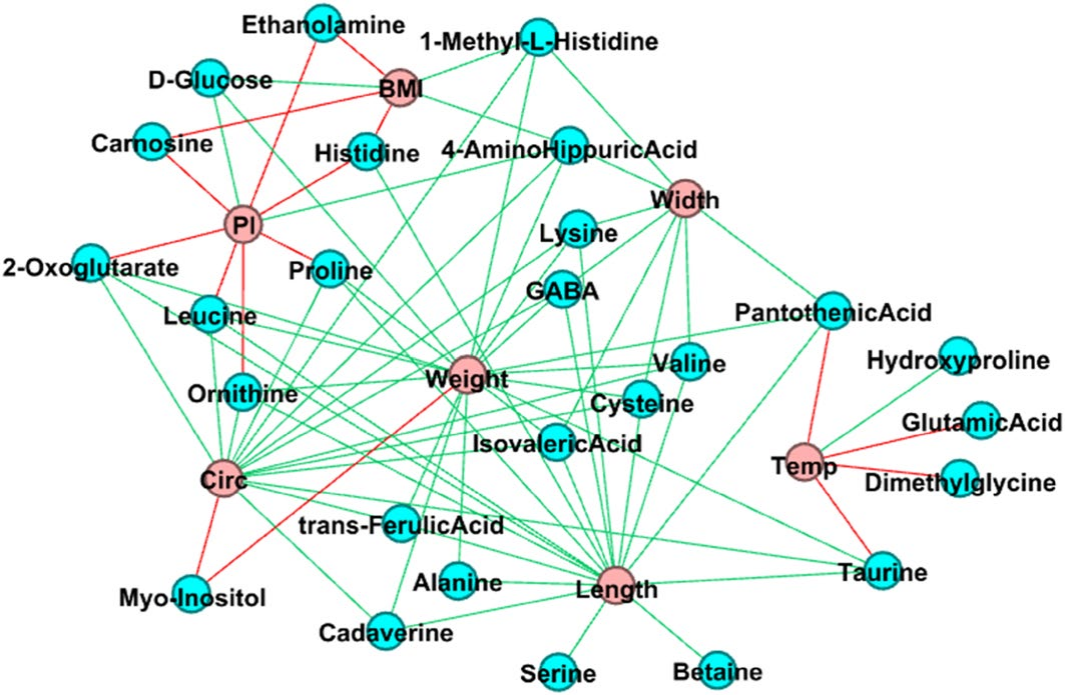
Bipartite graph between metabolites and biometric phenotypes of piglets. This graph was obtained after two separate sPLS (canonical mode, cutoff 0.45) analyses of each of the two metabolite datasets (AA and NMR, blue circles) with the phenotypic dataset (red circles). The two graphs were joined using Gephi. Connections between metabolites and phenotypes are in red for negative covariations and in green for positive covariations. *Temp* rectal temperature, *Circ* chest circumference, *BMI* Body Mass Index, *PI* Ponderal Index.

Multivariate analyses (sPLS, sparse Partial Least Squares) were performed to identify covariations between each dataset of metabolites and phenotypes, which are presented as an interaction graph (Fig. 3). These results highlighted that pantothenic acid and hydroxyproline had the highest VIP values for rectal temperature (Supplementary Table S2) and showed, respectively, negative and positive covariations with the rectal temperature. Similarly, BMI and PI were positively connected to 4-amino-hippurate, and BMI was negatively connected to carnosine. Many positive covariations were observed with birth weight, width between the two shoulders, chest circumference, and crown-to-rump length. In contrast, myo-inositol was negatively connected to birth weight and chest circumference.

## Discussion

Plasma metabolomic profiles of piglets were investigated at birth from umbilical cord blood. This blood comes from both the placenta and the fetus, thereby being influenced by maternal and fetal metabolisms. The non-targeted ^1^H-NMR metabolomic approach associated with the automatic identification and quantification of plasma metabolites allowed differentiating piglets according to their genetic origin. The use of these approaches also highlighted relationships between some metabolites and neonatal phenotypes that are known as potential indicators of piglet maturity at birth.

The genetic improvement of pig feed efficiency is a key to reduce feed costs and environmental impacts of pig production. Residual feed intake is a criterion for divergently selecting pigs for feed efficiency^15^. Selection for RFI induced differences in performance and physiology of growing pigs and sows (see reviews^4,16^). For the same growth rate, growing pigs from the LRFI line eat less than pigs from the HRFI line^16^, suggesting differences in nutrient partitioning, protein, and energy metabolism. The impact of selection for RFI on piglets is less described. In a study on more than 1000 litters, LRFI piglets were genetically lighter and more numerous at birth on average and grew faster up to weaning than HRFI piglets^4^. Moreover, a better ability to thermoregulate shortly after birth was suggested for LRFI piglets^6^. Similar differences were reported between Meishan and Large White piglets, the former being known as more mature at birth. Newborn Meishan piglets are as resistant to cold as Large White piglets despite being lighter^17,18^. Therefore, we hypothesized that maturity at birth might differ between the divergent lines selected for RFI. In the present study, LRFI piglets tended to be slightly shorter, at equal weight, and therefore stockier than the HRFI piglets, as shown by the morphology criteria width, BMI, and PI. The morphology of piglets is more important than birth weight to predict piglet survival until weaning^19,20^. It may thus be a better indicator of maturity at birth, thereby supporting a better maturity at birth of LRFI piglets.

Besides the modest differences in morphology, piglets from the two lines differed in plasma metabolome. In a previous study on the same lines, the use of an NMR metabolomics approach discriminated LRFI and HRFI growing pigs, with the most influential metabolites being AA (phenylalanine, glutamate, and tryptophan) and AA derivates^21^. The present study showed major differences for AA in newborn piglets as well. Concentrations of six AA (histidine, lysine, asparagine, aspartate, arginine, and ornithine) and one AA derivate (taurine) were 35% greater on average (11–62%) in HRFI than in LRFI piglets, while tryptophan concentrations were 16% lower. Moreover, more than a quarter of the other metabolites (18 out of 56) were influential and differential. Based on these metabolites, 11 enriched metabolic pathways were identified. Many of them were related to nitrogen and amino acid metabolism and less to energy metabolism.

One of the most clear-cut results concerns arginine, ornithine, aspartate, and 2-oxoglutarate. In cellular metabolism, the 2-oxoglutarate, also known as α-ketoglutarate, lies at the intersection between the carbon and nitrogen metabolism, connecting the tricarboxylic acid cycle (TCA or Krebs cycle, an energy-yielding cycle) to nitrogen assimilation reactions^22^. It is closely linked to the urea cycle, for which major intermediates are ornithine, arginine, and aspartate. The urea cycle might thus be more active in HRFI piglets. In a former study, the Krebs cycle has been also identified as having a greater activity in the HRFI pigs at 115 kg^23^. The urea cycle converts the ammonia derived from AA catabolism, extremely toxic, to non‐toxic urea for urinary excretion. Plasma concentrations of these metabolites being greater in HRFI than in LRFI piglets, the urea cycle might be more active in HRFI piglets. This could indicate more cycles of synthesis and degradation of AA. Alternatively, ornithine and arginine may be used as precursors for proline biosynthesis. In pigs, proline and its metabolite hydroxyproline make up to 10% of the collagen^24^, which is the main structural protein in the extracellular matrix of connective tissues and thus the most abundant protein in mammals. Nevertheless, it should be noted that proline and hydroxyproline did not participate in differentiating the two lines. Lastly, arginine, ornithine, aspartate, and asparagine belong to the arginine family of AA and these AA are interconvertible via interorgan metabolism in most mammals^25^. Besides being abundant in proteins, these AA also have key functions as regulators in nutrient metabolism and immune response, thereby affecting the efficiency of feed, including in pigs^21,25^. Arginine is also known to stimulate protein synthesis and body growth by stimulating the activity of the mammalian target of rapamycin (mTOR), and arginine deficiency is a limiting factor for the growth of sow-reared piglets^25,26^. Greater concentrations of plasma arginine in HRFI piglets do not seem consistent with the lower growth rate of these piglets up to weaning. This indicates the need to further investigate the growth trajectories of the piglets from the two lines, altogether with their metabolic signatures, to better understand the role and evolution of these metabolites after birth.

The greater concentrations of plasma tryptophan in pigs of the LRFI line observed here at birth were previously observed in LRFI growing pigs at 15 and 23 weeks of age^27^. On the same lines, the tryptophan has been identified as one of the blood metabolic indicators explaining the variability of growth for 12-week-old pigs^28^. Tryptophan is a component of body proteins and a precursor of serotonin, and its metabolism is closely related to immune and inflammatory responses^29^. In several mammalian species including pigs and humans, inflammatory conditions stimulate the catabolism of tryptophan to kynurenine via the activation of indoleamine 2,3-dioxygenase enzyme^29^, which enhances the synthesis of picolinic acid and two neuroactive metabolites, namely kynurenic and quinolinic acids^30^. Interestingly, LRFI piglets presented lower plasma concentrations of these three metabolites along with a greater concentration of tryptophan, compared to the HRFI piglets. These findings indicate that piglets from the two lines selected for RFI differed in tryptophan metabolism, which may impact the regulation of growth and the immune function.

Besides these major differences in nitrogen and specific amino acid metabolism, modest differences in energy metabolism between LRFI and HRFI piglets were also shown. The greater concentrations of lactate and pyruvate of LRFI piglets at birth may be used for gluconeogenesis. The piglet at birth has a very low energy reserve and very high requirements for physical activity (to reach the udder and suckle) and for thermoregulation^31^. To meet these needs and therefore to survive after birth, the piglet relies on the energy supplied from its glycogen reserve and colostrum. A greater ability for gluconeogenesis might be an advantage for post-natal performance. Differences in energy metabolism were found between the lines before, more systematically than the protein related pathways^16^.

Piglets from the two lines also differed by their plasma concentrations of betaine and cadaverine, which were greater in HRFI piglets than in LRFI piglets (VIP scores > 2). Betaine, also named glycine betaine or trimethylglycine, is a methyl donor. Methyl donors are important for liver function, cellular replication, and detoxification reactions^32^. Cadaverine is one of the polyamines that can be synthesized by the porcine placenta and that plays a role in DNA and protein synthesis^33^. Taurine and pantothenic acid (also known as vitamin B5) were also two influential and differential metabolites. Taurine is known as a regulator of oxidative stress and fetal taurine is critical for neurological and cardio-vascular development^24,34^. Moreover, dietary taurine supplementation to gilts during late gestation and lactation improved the growth performance of piglets until weaning, possibly by improving the antioxidant ability, intestinal morphology, and barrier function of piglets^35^. Pantothenic acid is the key precursor for the biosynthesis of CoenzymeA and thereby plays an essential role in the general metabolism of carbohydrates, fats, and proteins^36^. It is also a precursor of beta-alanine (and vice-versa) and, consistently, the beta-alanine metabolism pathway was identified as one of the pathways differentiating the two lines. However, it is not possible to know whether greater concentrations of these four metabolites may give an advantage to HRFI piglets over LRFI piglets or whether, on the contrary, they reflect a lower use or efficiency of these metabolites.

Regardless of piglet lines, covariations were observed between piglet phenotypes at birth and metabolites. Plasma concentrations of myo-inositol were negatively associated with piglet birth weight and chest circumference. Myo-inositol is the most prominent stereoisomer of inositol and the structural basis for many secondary messengers. In particular, it plays a major role in insulin action and glucose metabolism^37^. It has been proposed as a marker of intra-uterine growth retardation in humans and pigs^37,38^. We also showed that myo-inositol concentrations decreased in pig fetuses during the last month of gestation and were lower in the most mature genotype (Meishan versus Large White pigs^12^). Myo-inositol may thus be an indicator of pig (im)maturity at birth. Our previous findings also suggested that plasma concentration of proline might be another indicator of piglet maturity^12^. Consistently, positive covariations and VIP > 1 were observed between proline concentrations and piglet phenotypes at birth (weight, length, circumference, and PI) in the present study.

Interestingly, the rectal temperature of piglets at birth co-varied with few metabolites, less than other phenotypic traits. Rectal temperature at birth and piglet ability to maintain body temperature after birth are of the utmost importance for colostrum consumption and short-term survival after birth. The strongest covariations were observed with hydroxy-proline (positive), pantothenic acid, and taurine (negative covariations). Although pantothenic and taurine were influential and differential metabolites for the genetic lines, the rectal temperature at birth did not differ between LRFI and HRFI in the present study, consistently with our previous findings^6^. During the first hour after birth, however, HRFI piglets had lower ear tip temperatures than LRFI which suggested differences in thermoregulation abilities^6^.

## Conclusion

Our study provides new insights into the biological differences related to the selection on RFI in newborn piglets. Major differences in nitrogen and amino acid metabolisms were highlighted, especially related to the arginine family of AA and to the tryptophan metabolism. Modest differences in energy metabolism were in favor of the LRFI piglets and better neonatal thermoregulation abilities. Myo-inositol and proline, two metabolites previously proposed as potential markers of maturity of piglets, did not differentiate piglets for RFI. However, here, covariations observed between piglet weight and morphology and myo-inositol and proline support these two metabolites as potential markers of maturity.

## Methods

### Genetic design and animal management

#### Ethics statement

This study was conducted in accordance with the French legislation on experimentation and ethics. The French Ministry of Research and Innovation authorized this experiment on living animals at the INRAE facilities UE1372 GenESI Genetics, Pig phenotyping and Innovative breeding facility (Surgères, France, https://doi.org/10.15454/1.5572415481185847E12) with the agreement number APAFiS 13648-2018020417291866 v4. In these conditions, this study follows the ARRIVE guidelines (Animal Research: Reporting of In Vivo Experiments), and is committed to the 3Rs of laboratory animal research and consequently used the minimal number of animals to reach statistical significance.

#### Animals and experimental design

The experiment was conducted in two batches in January and June 2018. It involved 97 piglets: 51 LRFI piglets born from 6 LRFI sows and 46 HRFI piglets born from 7 HRFI sows, balanced in the two batches. These piglets represented the tenth generation of the genetic lines. Sows were inseminated with 4 HRFI boars and 5 LRFI boars to capture most of the line variability. Piglets were born in free farrowing pens (300 × 246 cm), in which sows were crated at the time of birth^7^ and then, free in their pen until weaning. A heat lamp (Interheat Inc., Seongnam-si, Korea; 175 W) was suspended in the pen, on the side of the sow. Piglets had access to a creep area (180 × 90 cm).

#### Blood sampling and measurements

At birth, each piglet was immediately dried and blood (5 mL) was collected by dripping from the umbilical cord in heparinized tubes. Plasma was prepared by low-speed centrifugation (2000*g* for 10 min at 4 °C) and stored at − 80 °C until analysis. Just after blood sampling, various measures were recorded as previously reported in Schmitt et al.^6^. Piglets were weighed (Weight) and their body length (crown-to-rump, Length), shoulder width (Width), and chest circumference were measured. Piglet body mass index (BMI, birth weight/crown-to-rump length^2^) and ponderal index (PI, birth weight/crown-to-rump length^3^) were calculated. Rectal temperature (Temp) was measured at birth with a rectal thermometer (SC12; SCALA Electronic GmbH, Stahnsdorf, Germany). These phenotypic measures composed in the so-called “PHENO” dataset.

### Metabolites quantification

#### ^***1***^***H NMR protocol***

The protocol was fully described in Lefort et al.^12^ for the same samples. Here, the main steps of the spectra acquisition and analysis are recalled. Each plasma sample (200 µL) was diluted in 500 µL phosphate buffer prepared in deuterated water (0.2 M, pH 7.0) containing TSP (1.17 mM) as internal standard, vortexed, centrifuged at 5000*g* for 15 min at 4 °C, and 600 µL of the supernatant were transferred into 5 mm NMR tube. All ^1^H NMR spectra were acquired on a Bruker Avance III HD NMR spectrometer (Bruker Biospin, Rheinstetten, Germany) operating at 600.13 MHz for 1H resonance frequency and at 300 K, using the Carr-Purcell-Meiboom-Gill (CPMG) spin-echo pulse sequence. Spectrum preprocessing (group delay correction, solvent suppression, apodization, Fourier transformation, zero-order phase correction, internal referencing, baseline correction, and window selection) was performed using the package PepsNMR^39^ (version 1.2.1) with the TSP peak for internal reference. Finally, spectra were aligned with each other using the method implemented in the ASICS package^12,13^. The metabolites in the NMR spectra were quantified using the ASICS package with default procedure and parameters, except for the threshold under which the signal is considered as noise, which was set to 0.01, and the multiplicative and additive noise standard deviations, which were set to 0.07 and 0.09, respectively. The NMR raw spectra are available in the Metabolights database under accession number MTBLS2137. A raw complex spectrum with the spectrum reconstructed by ASICS from its deconvolution method is presented in see Supplementary Figure S1 and was manually annotated for ten metabolites identified by ASICS. This plot shows that the original spectrum is well reconstructed by ASICS, which is a good indication of the quality of the quantification.

#### UPLC protocol

Amino acid concentrations of the same plasma samples were determined by Ultra Performance Liquid Chromatography (UPLC). Plasma AA concentrations were obtained using an ultra HPLC system (Waters Acquity UPLC system, Waters, Guyancourt, France) coupled to an Acquity tunable UV detector and a mass detector (SQD detector) to identify the few coeluting chromatographic peaks. The column was a MassTrak AAA column (2.1 times 150 mm). Amino acid derivatization was performed using an AccQ·Tag Ultra derivatization (MassTrak AAA Waters, Milford, MA). Norvaline was used as an internal standard and a mixture of amino acids was used for calibration and quantification. The mixture contained 42 amino acids and related compounds, each of them being provided at a known concentration. The mixture was analysed 3 times and the % deviation for each amino acid should not exceed 3%. The equation of the calibration curve was then calculated on the average of the 3 analyses and used to compute the unknown concentrations. The Empower 2 chromatography software (Waters Corporation, Milford, MA, USA) was used for instrument control and data acquisition.

### Statistical analyses

All statistical analyses were carried out with R^40^ (version 4.0.3).

#### Data cleaning

The data contained a few missing values: seven in the PHENO dataset (see Supplementary Table S1 for details) and two for two different AA (hydroxyproline and sarcosine). These missing values were imputed with a PCA based method (independently for the two datasets) using the R package missMDA^41^ prior PLS analyses. For the other analyses (correlation computation and statistical tests), missing values were ignored (dropped from data prior computation).

Principal Component Analysis were performed in each dataset (PHENO, AA, ASICS) independently to search for outliers or batch effects. Only the AA dataset showed two outliers (in HRFI piglets) and were removed prior multivariate analyses, following the ropls package recommendation.

#### Correlation analysis

Spearman correlations were computed between metabolites quantification with NMR and UPLC dosage of the same metabolites.

#### Multivariate analyses of association with phenotypes

First, multivariate analyses were performed to assess the predictive power of groups of metabolites on phenotypes, and to find groups of metabolites and phenotypes co-varying.

First, the predictive power of metabolites on each of the phenotypes was assessed using orthogonal partial least squares (OPLS) as implemented in the R package ropls^42^ (version 1.20.0). More precisely, each of the two metabolite datasets (AA and ASICS) was, in turn, used to predict the genetic line as well as every phenotype (independently). Binary phenotypes (genetic line or survival) were predicted using OPLS-DA, and numeric phenotypes (birth weight—weight0, crown-to-rump length—Length, shoulder width—Width, chest circumference—Circ, and rectal temperature—Temp) were predicted using OPLS. Influential metabolites were chosen as those with a VIP (Variable Importance in Projection) value larger than 1 (commonly chosen arbitrary value). In addition, the model relevance was assessed by testing the optimal number of predictive (predI) and orthogonal components (orthoI) together, using permutation tests at the 0.05 level (permI = 1000).

Second, the R package mixOmics (http://mixomics.org/) was used to identify groups of metabolites covarying with groups of phenotypes. More precisely, two sparse PLS (sPLS, mode "canonical") were performed with, respectively, AA and ASICS as the first dataset and with the morphometric values measured on the same neonates as the second dataset. The most interesting covariations identified by the method were further selected using a correlation cutoff of 0.45. They were used to build two networks of highly covarying variables, one for each analysis (with AA or ASICS as the first dataset). In these networks, links between variables corresponded to a common selection on the same axis of the sPLS. Finally, the two networks were joined with the Gephi application (version 0.9.2, https://gephi.org/).

#### Differential analysis

To confirm the statistical significance of found influential variables, several tests were performed to identify metabolites whose dosages or ASICS quantifications were significantly different between the two genetic lines or phenotypes significantly different between the two lines. First, non-parametric Wilcoxon tests were performed for each variable independently and then, a mixed-effect model with a fixed line effect and the random sow effect nested in the line effect (R packages nlme version 3.1-149 and multcomp version 1.4-14) was fitted. *P-*values were adjusted with the Benjamini–Hochberg^43^ procedure to control the false discovery rate (FDR) in each of the three datasets independently. Variables were considered significant at the threshold FDR < 0.05.

Note that we could not expect much from the mixed-effect model with the random sow effect since the genetic line effect is strongly related to this effect. It was nevertheless performed to assess if any of the found effect could be strong enough to be visible even after the sow effect has been accounted for.

Finally, all metabolites from the ASICS datasets that were found significant were manually confirmed by the identification of their corresponding peaks in the spectrum (see Supplementary Fig. S1). In addition, the peaks leading to the identification of all metabolites from the ASICS datasets that were found significant or that had a VIP > 1 in one of the PLS/OPLS-DA analyses (results provided in Supplementary Table S1 and Supplementary Table S4) were systematically positioned on the spectrum (Supplementary Table S4).

### Metabolic pathway analyses

The HMDB (Human Metabolome Database, https://hmdb.ca/) and KEGG^44^ (Kyoto Encyclopedia of Genes and Genomes, www.genome.jp/kegg/) databases were used to recover molecular information for each metabolite. The KEGG mapper module was used to identify metabolic pathways including identified metabolites. To ensure the strength of our analysis, we performed the enrichment only with the most confirmed metabolites (i.e., the 18 metabolites that were found both differential and influential in the ASICS dataset and have all been confirmed on the spectrum—see Supplementary Fig. S1—together with the 7 metabolites found influential in the AA dataset).

Metabolite Set Enrichment Analysis was performed with MetaboAnalyst 5.0 exploring SMPDB^45^ database with the Human genome as a reference. To verify the high similarity of metabolic pathways between the two genomes, human and pig genomes were used as a reference. As more information is available for the human genome, results were considered with the human genome as a reference.

## Supplementary Information

Supplementary Figure S1.

Supplementary Table S1.

Supplementary Table S2.

Supplementary Table S3.

Supplementary Table S4.

## Data Availability

The datasets (NMR raw spectra) generated during the current study are available in the Metabolights repository under accession number MTBLS2137.
