# Supplementary material for: Plasma 1H-NMR metabolic and amino acid profiles of newborn piglets from two lines divergently selected for residual feed intake
Source: Sci Rep. 2023 May 2;13:7127. doi: 10.1038/s41598-023-34279-5 (PMC10154392; doi:10.1038/s41598-023-34279-5)
Supplement: Supplementary file 1 — Supplementary Figure S1. [file 41598_2023_34279_MOESM1_ESM.pdf]

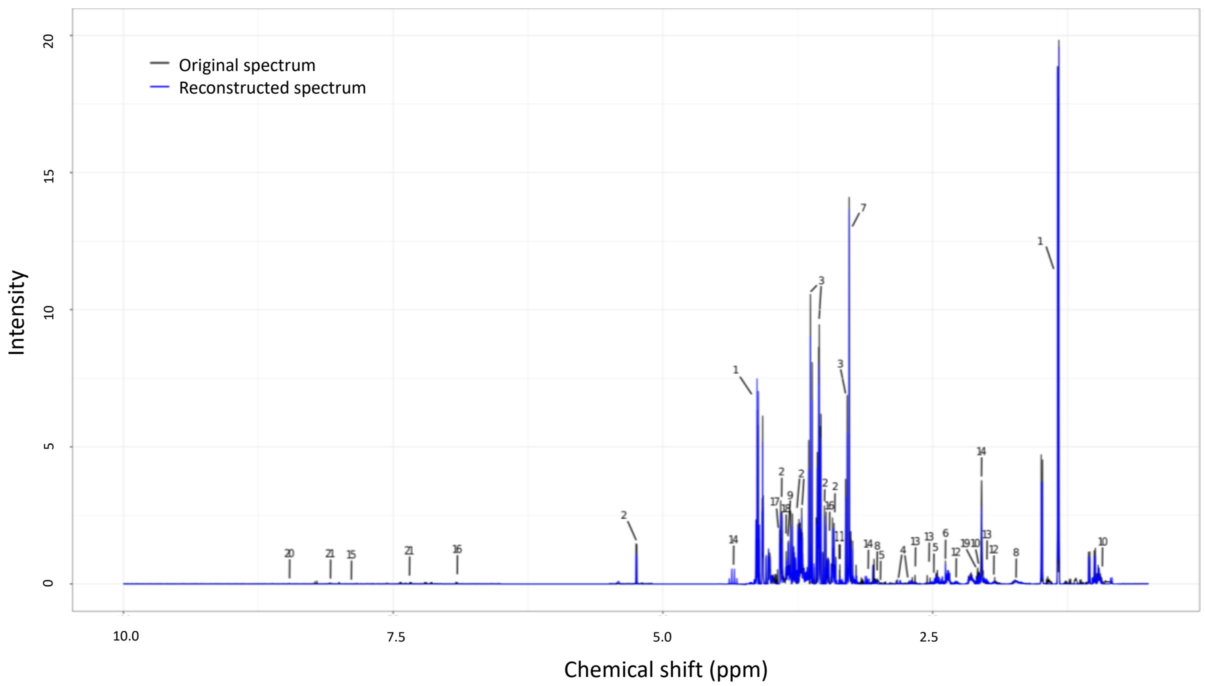

Figure S1. Raw and reconstructed spectra (the latter as obtained from the quantification estimated by ASICS) from one sample of the present dataset. Numbers correspond to: 1, Lactate; 2, D-Glucose; 3, Myo-Inositol; 4, L-Aspartate; 5, 2-Oxoglutarate; 6, Pyruvic Acid; 7, Betaine; 8, Cadaverine; 9, Guanidinoacetic Acid; 10, Pantothenic Acid; 11, Methanol; 12, GABA; 13, N-Acetyl-L-Aspartic Acid; 14, S-Acetamidomethylcysteine; 15, 2-Picolinic Acid; 16, Homovanillic Acid; 17, trans-Ferulic Acid; 18, Adenosine; 19, Isovaleric Acid; 20, Quinolinic Acid; 21, Kynurenic Acid.
